# BRAF Mutation Is Associated with Hyperplastic Polyp-Associated Gastric Cancer

**DOI:** 10.3390/ijms222312724

**Published:** 2021-11-25

**Authors:** Rina Fujiwara-Tani, Ayaka Okamoto, Hiroyuki Katsuragawa, Hitoshi Ohmori, Kiyomu Fujii, Shiori Mori, Shingo Kishi, Takamitsu Sasaki, Chie Nakashima, Isao Kawahara, Yudai Hojo, Yukiko Nishiguchi, Takuya Mori, Takeshi Mizumoto, Kenta Nagai, Yi Luo, Hiroki Kuniyasu

**Affiliations:** 1 Department of Molecular Pathology, Nara Medical University, 840 Shijo-cho, Kashihara 634-8521, Nara, Japan; rina_fuji@naramed-u.ac.jp (R.F.-T.); dc117023@naramed-u.ac.jp (A.O.); mol.path.hk@gmail.com (H.K.); brahmus73@hotmail.com (H.O.); toto1999-dreamtheater2006-sms@nifty.com (K.F.); m.0310.s.h5@gmail.com (S.M.); nmu6429@yahoo.co.jp (S.K.); takamitu@fc4.so-net.ne.jp (T.S.); c-nakashima@naramed-u.ac.jp (C.N.); isao_kawahara@a011.broada.jp (I.K.); yudaihojo@outlook.com (Y.H.); yukko10219102@yahoo.co.jp (Y.N.); pt_mori_t@yahoo.co.jp (T.M.); 2 Miyoshi Central Hospital, 10531 Higashi-Sakaya-cho, Miyoshi 728-8502, Japan; T.Mizumoto@city.miyoshi.hiroshima.jp (T.M.); K.Nagai@city.miyoshi.hiroshima.jp (K.N.); 3 Key Laboratory of Neuroregeneration of Jiangsu and Ministry of Education, Co-Innovation Center of Neuroregeneration, Nantong University, Nantong 226001, China

**Keywords:** gastric hyperplastic polyp, gastric cancer, BRAF mutation, *H. pylori*, oxidative stress

## Abstract

Gastric hyperplastic polyps (GHP) are frequently found to be benign polyps and have been considered to have a low carcinogenic potential. The characteristics of the hyperplastic polyp-associated gastric cancer (HPAGC) remain unclear. Therefore, we analyzed samples from 102 GHP patients and identified 20 low-grade atypical GHPs (19.6%), 7 high-grade atypical GHPs (6.9%), and 5 intramucosal cancer samples (4.9%). GHP atypia was more common in the elderly and increased with increasing polyp size. In particular, polyps larger than 1 cm were associated with a higher grade and cancer. Furthermore, mucus production decreased with increasing atypia. Although no correlation was found between atypia and Helicobacter pylori infection or intestinal metaplasia, enhanced proliferative ability (Ki-67) did correlate with atypia, as did nuclear 8-hydroxy-2’-deoxyguanosine levels. Interestingly, 4-hydroxynonenal levels in granulation tissue and the area ratio of granulation tissue within polyps also correlated with GHP atypia. In five cases of HPAGC, three cases exhibited caudal type homeobox transcription factor (CDX2)-positive cells and a mixed mucin phenotype, which is considered to be related to *H. pylori* infection. By contrast, two cases were CDX2 negative, with a gastric mucin phenotype, and *H. pylori* infection was not observed in the tumor or the surrounding mucosa. In these cases, a v-raf murine sarcoma viral oncogene homolog B1 (BRAF) mutation (V600E) was detected. All cancer samples showed high stemness and p53 protein accumulation, but no KRAS mutations. The molecular and phenotypic characteristics of the cases characterized by BRAF mutations may represent a novel subtype of HPAGC, reflecting a conserved pathway to oncogenesis that does not involve *H. pylori* infection. These findings are worthy of further investigation in a large-scale study with a substantial cohort of HPAGC patients to establish their clinical significance.

## 1. Introduction

Gastric cancer is currently the third most common cause of cancer-related death in Japan [1]. Inflammation is a key feature of gastric cancer, and oxidative stress due to chronic inflammation caused by *Helicobacter pylori* plays an important role in the carcinogenesis of gastric cancer [2]. Chronic atrophic gastritis and intestinal epithelialization are known to be precancerous conditions caused by *H. pylori* [3]. In our previous study, we showed that the activation of oxidative stress and v-akt murine thymoma viral oncogene homolog (AKT) increased substantially in each of the following categories of chronic gastritis: chronic gastritis without *H. pylori*, chronic active gastritis with *H. pylori*, chronic metaplastic gastritis without *H. pylori*, and chronic gastritis with atypia without *H. pylori* [4]. This is due to the shortening of telomeres and activation of telomerase reverse transcriptase, which are associated with repeated mucosal regeneration due to chronic inflammation [5] and are thought to lead to hyperplasia of gastric mucosal stem cells [6,7].

Gastric hyperplastic polyps (GHPs) are the most common polyps encountered in the stomach (along with fundic gland polyps) and are detected in 1.9% of 110,000 patients subjected to gastroscopy [8]. Patients with GHPs are usually asymptomatic; however, some may present with dyspepsia, heartburn, abdominal pain, anemia, or upper gastrointestinal bleeding [9]. GHPs are thought to occur during the repair of damaged mucosa [9]. Malignant transformation of GHP is very infrequent, with an incidence of 0.3–3% [10,11]. In previous research, hyperplastic polyp-associated gastric cancer (HPAGC) is derived from dysplastic foci in GHP [12,13]. However, the histological and molecular pathological markers for its definitive diagnosis have not been established.

Recently, genome and proteome analyses of gastric cancer have been performed as part of The Cancer Genome Atlas (TCGA) project, and it has been established that gastric cancer can be classified into four molecular subtypes [14]: (1) Epstein–Barr virus, (2) microsatellite instability, (3) genomically stable, and (4) chromosomal instability subtypes. Such comprehensive molecular pathological analysis provides a better understanding of the development of gastric cancer and enables the extraction of molecular targets that lead to drug discovery. However, due to the scarcity of cases, the details of HPAGC have not been completely clarified. Doing so may improve our understanding of this specific type of gastric cancer and provide insight as to whether different treatment approaches are required to deal with this cancer. In this study, we investigated the characteristics of early HPAGC and analyzed its underlying pathogenic mechanisms and molecular abnormalities.

## 2. Results

### 2.1. GHP Atypia and the Associated Clinical and Morphological Characteristics

**Figure 1:**
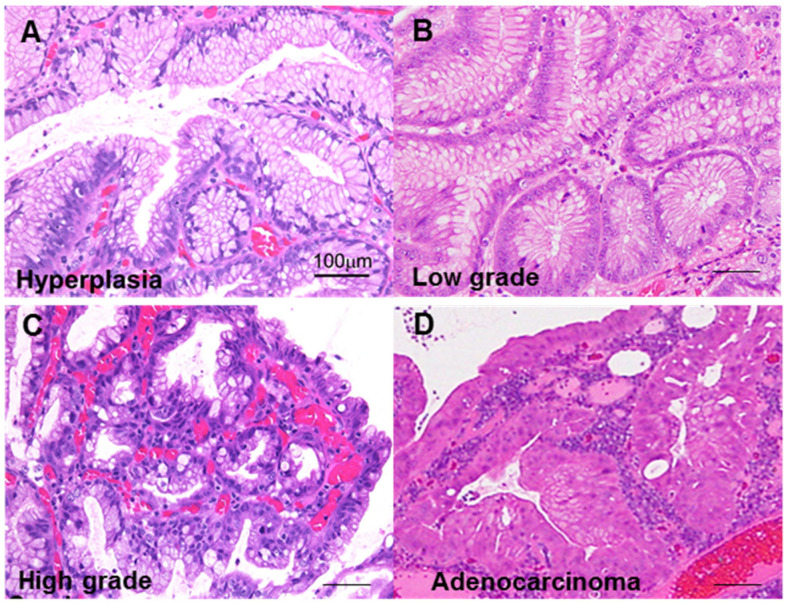
Atypia in hyperplastic polyps. (**A**) Hyperplastic polyp without atypia. (**B**) Low-grade atypia, notable mild nuclear swelling. (**C**) High-grade atypia, notable moderate to marked nuclear swelling, nuclear pseudostratification, and decrease in mucus production. (**D**) Carcinoma in hyperplastic polyp (well-differentiated tubular adenocarcinoma), marked nuclear swelling, nuclear pseudostratification, decrease in mucus production, and structural atypia. Scale bar, 100 μm.

First, we classified GHP atypia into four categories: non-atypical GHP with no nuclear and no structural atypia with abundant mucus production; low-grade atypia with mild to moderate nuclear swelling; high-grade atypia with moderate to prominent nuclear swelling, pseudostratified nuclei, and decreased periodic acid–Schiff (PAS)-positive mucus production; and HPAGC with the cellular characteristics of high-grade atypia as well as structural atypia and/or invasion (Figure 1).

**Figure 2:**
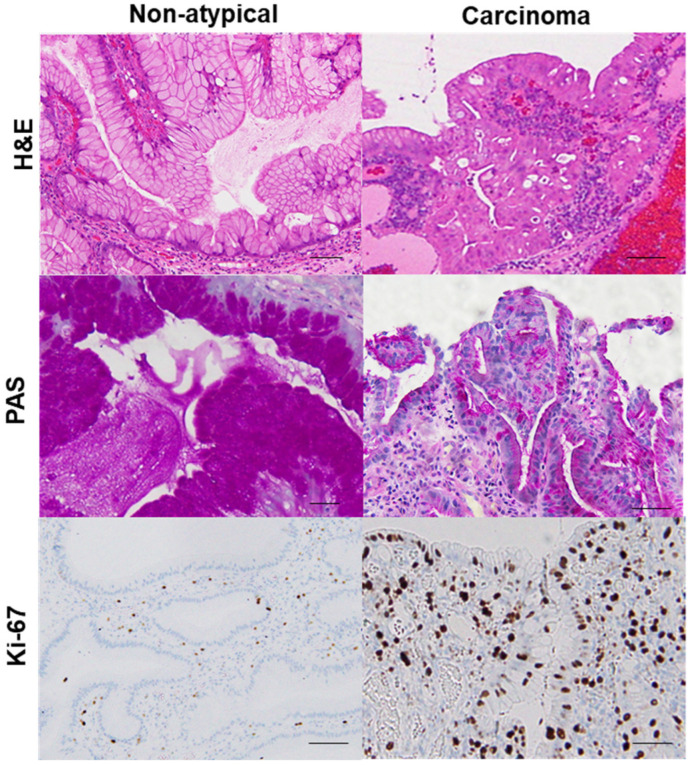
Comparison of mucus production and proliferative activity between a hyperplastic polyp and its carcinoma lesion.In the same hyperplastic polyp (case 2 in Table 3), non-atypical regions and the carcinoma lesion were compared. In the non-atypical region, PAS-positive mucus is abundant in most foveolar epithelial cells, whereas in the cancer lesion, PAS-positive mucus is only found in a few cells. Ki-67-positive cells were scattered in non-atypical regions with an index of 5%. By contrast, in the cancer lesion, a high number of Ki-67-positive cells were evident, up to the surface layer of the glands (index of 92%). H&E, hematoxylin and eosin; PAS, periodic acid–Schiff.

Using this categorization process to sort the samples from 102 GHP patients, we identified 20 GHP samples as low-grade atypia, 7 GHP samples as high-grade atypia, and 5 GHP samples as HPAGC (Table 1). Atypical GPHs were more common in the elderly and the incidence increased with an increase in polyp size. In particular, polyps larger than 1 cm were associated with a higher grade and cancer. In addition, mucus production decreased with increasing atypia. By contrast, no correlation was observed between atypia and *H. pylori* infection or intestinal metaplasia. Immunohistochemistry for Ki-67 expression showed that enhanced proliferative activity also correlated with atypia (Figure 2).

### 2.2. GHP Atypia and Oxidative Stress

**Figure 3:**
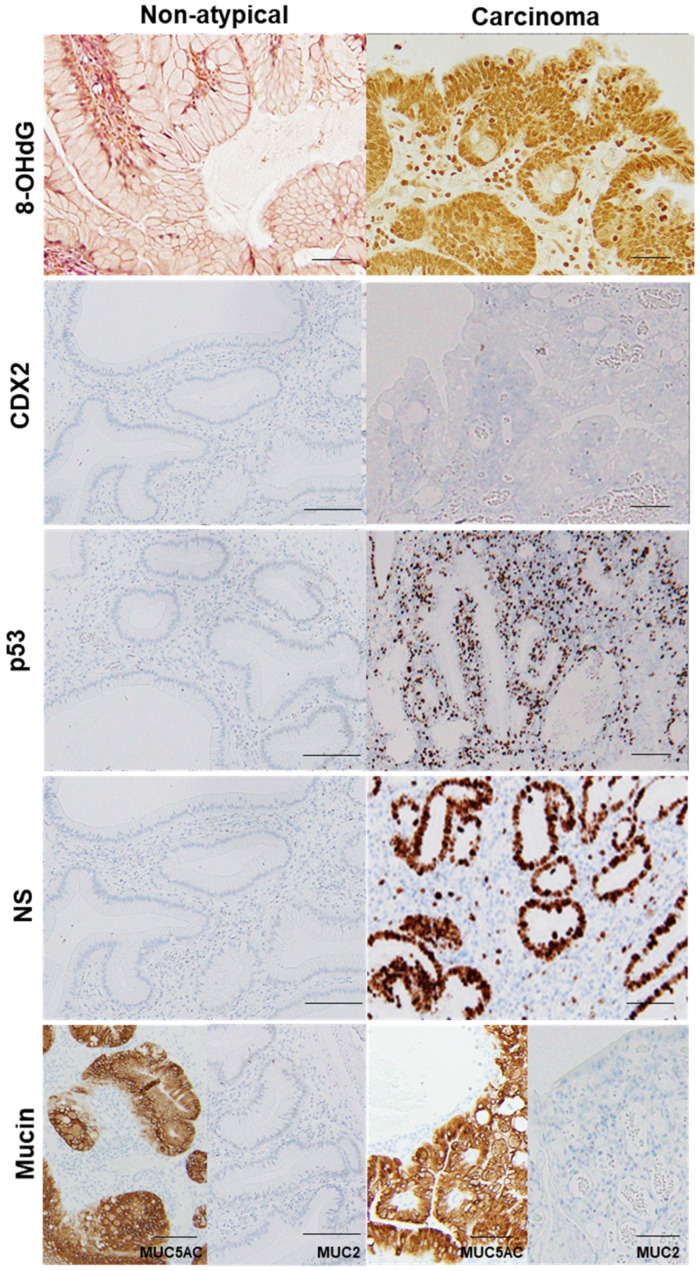
Comparison of oxidative stress, intestinal phenotype, and stemness between hyperplastic polyp and its carcinoma lesion.

Oxidative stress has a prominent influence on gastric carcinogenesis [2]. When we investigated the relationship between atypia and oxidative stress in GHPs (Figure 3, Table 2), we found a correlation between atypia and nuclear 8-hydroxy-2′-deoxyguanosine (8-OHdG) levels. Interestingly, 4-hydroxy nonenal (HNE) levels in the granulation tissue of GHPs also correlated with atypia, as did the area ratio of granulation tissue within the polyps. Nuclear 8-OHdG levels and granulation tissue area, or 4-HNE levels in granulation tissue, were all correlated (Pearson r = 0.6489 and 0.7245, *p* < 0.0001 and < 0.0001, respectively). These results suggest that granulation tissue may play a role in generating oxidative stress in GHP, and it may be a trigger for GHP carcinogenesis.

In the same hyperplastic polyp (case 2 in Table 3), non-atypical regions and the carcinoma lesion were compared. 8-OHdG in the nuclei was scattered in non-atypical regions (index of 4%), whereas the cancer lesions exhibited a substantial number of positive cells (index of 100%). Both non-atypical regions and the cancer lesion showed no nuclear expression of CDX2. p53 nuclear accumulation was found in the cancer lesion (index of 72%), but not in non-atypical regions (index of 0%). Nuclear staining for NS was only observed in a few cells in non-atypical regions (index of less than 1%), whereas cancer lesions showed high labeling of NS (index of 87%). 8-OHdG, 8-hydroxy-2′-deoxyguanosine; CDX2, caudal type homeobox transcription factor 2; NS, nucleostemin.

### 2.3. GHP Atypia and Expression of Cancer-Associated Proteins

We next examined atypia in GHP samples and the expression of cancer-associated proteins, namely caudal type homeobox transcription factor 2 (CDX2), p53, and nucleostemin (NS) (Figure 3, Table 2). CDX2, which is associated with the acquisition of the intestinal phenotype associated with *H. pylori* infection [17], did not correlate with atypia (similarly to the results for *H. pylori* and intestinal metaplasia). By contrast, p53 expression was negative in non-atypical GHPs and GHPs with low-grade atypia, but positive for 43% of high-grade GHP samples and 100% positive for the HPAGC samples. In addition, the number of cells that were positive for NS (a stem cell marker) increased in correlation with atypia.

### 2.4. Features of HPAGC

**Figure 4:**
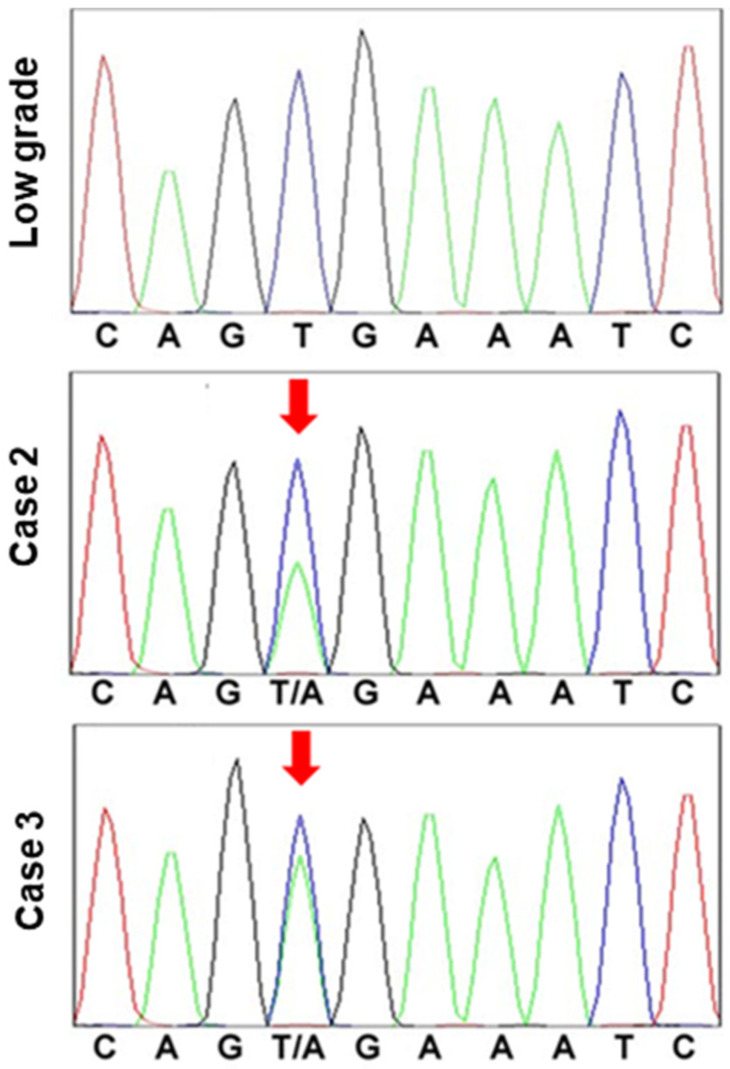
*BRAF* mutation in hyperplastic polyp carcinoma.

Finally, we examined the five cases in which cancer was found in the polyps (Table 3). In these cases, the size of the polyp exceeded 1 cm in four of the five cases. All cancer lesions were 4 mm or less intramucosal cancers with a well-differentiated histology and were carcinomas in situ, except for one case. Two cases exhibited CDX2-positive staining in ≥10% of the cells. One case had CDX2-positive cells in 5% of the cells, whereas the other two were negative for CDX2. Each of the three CDX2-positive cases was also characterized by a mixed mucin phenotype, whereas the two CDX2-negative cases exhibited a gastric mucin phenotype. There was no correlation between polyp size and p53 or NS indices, histology, CDX2 expression, or mucin type; and all of the cases were negative for *KRAS* G12D/G13D mutations. By contrast, *BRAF* V600E mutation was found in the two cases which exhibited a gastric mucin phenotype (Figure 4).

*BRAF* exon 15 was sequenced in a low-grade atypia polyp and hyperplastic polyp-associated gastric cancer (cases 2 and 3). In the low-grade atypia polyp, codon 600 exhibited the normal sequence, G-T-G (Val). By contrast, in the cancer cases, codon 600 was determined to be mutated, with the sequence G-T/A-G (Val/Glu).

## 3. Discussion

In the present study, we analyzed the samples from 102 patients with GHPs. Intramucosal cancer was detected in five GHP cases (4.9%). In these HPAGCs, CDX2-positive cells were found in three cases that also presented with mixed mucin phenotype, which is considered to be related to *H. pylori* infection. By contrast, two cases were CDX2-negative and exhibited a gastric mucin phenotype, and there was no evidence of *H. pylori* infection in the tumor or the surrounding mucosa. Although all of the HPAGC cases demonstrated p53 protein accumulation and high expression of a stem cell marker, it was particularly interesting that *BRAF* mutations were identified in two of the five cases.

*H. pylori* and type A gastritis are associated with 89% of GHPs [18], and it is believed that GHPs develop during the repair of damaged mucosa [9]. The eradication of *H. pylori* has been reported to reduce the incidence of GHPs [19]. However, even if *H. pylori* infection is demonstrated in the mucosa of the non-polyp region, *H. pylori* is not detected in the GHPs in one-third of the patients [20]. It was reported that the *H. pylori* infection rate in patients with GHPs (excluding polypoid foveolar hyperplasia and gastric mucosal prolapse polyps) was 21% [21]. This incidence is approximately equivalent to our data. In addition, although the *H. pylori* infection rate has declined in recent years, the prevalence of GHPs has increased, raising questions about *H. pylori* infection as the cause of GHPs [8]. CDX2 is a transcription factor that causes intestinal differentiation and it is expressed during gastric carcinogenesis with *H. pylori* infection, resulting in the acquisition of an intestinal phenotype and an increase in stemness [17,22,23]. In this study, atypical GHP was not correlated with *H. pylori* infection in the atypical lesion and the surrounding mucosa, intestinal metaplasia, or CDX2 expression. These findings suggest that, unlike non-polyp-derived gastric cancer, *H. pylori* infection is unlikely to be the main cause of HPAGC.

In non-polyp-derived gastric cancer, *H. pylori* infection induces genetic and epigenetic abnormalities [24,25] as a result of intracellular signal disruption and induction of genetic instability by the CagA protein [26] and the activation of oxidative stress [27]. By contrast, the results from the current study indicated that increased GHP atypia did not correlate with *H. pylori* infection, whereas 8-OHdG levels in atypical cells and the amount of granulation tissue in polyps or 4-HNE levels in granulation tissue did correlate with increased atypia. Moreover, these markers of oxidative stress also correlated with each other; however, *H. pylori* infection did not correlate with 8-OHdG or 4-HNE levels. These results suggest that inflammatory granulation tissue may be a source of oxidative stress in GHP, promoting genetic abnormalities associated with carcinogenic pathways. The association between inflammatory granulation tissue and carcinogenesis has been demonstrated using a foreign body carcinogenesis model, in which the generation of oxidative stress in foreign body granulation tissue was shown to be a key factor in cancer development [28]. The factors responsible for the formation of inflammatory granulation tissue in GHP are unclear. There is a morphological similarity between GHPs and cap polyps in the large intestine [29,30]. Because cap polyps are caused by mechanical stimuli such as mucosal prolapse syndrome [31], mechanical stimuli associated with gastric motility might be involved in the formation of inflammatory granulation tissue in GHP.

Another characteristic finding in this study was the presence of a *BRAF* mutation in HPAGC. Of the five HPAGC cases, two possessed the *BRAF* V600E mutation and a gastric mucin phenotype. BRAF is a downstream effector of KRAS, and its prognostic value in colorectal cancer is widely accepted [32]. However, *BRAF* mutations occur at a very low frequency in gastric cancer, with only 2.9% of screened cases exhibiting a *BRAF* mutation [33]. Similarly, *KRAS* mutation frequency is also very low in advanced gastric cancer (4.9%), even in a report showing a comparatively high frequency [34]. These results indicate that, unlike in colorectal cancer, the oncogenic activation of mitogen-activated protein kinases by driver mutations is uncommon in gastric cancer. By contrast, the *phosphatidylinositol 3-kinase catalytic subunit alpha (PIK3CA)* mutation (which also occurs at a low frequency) was present in 5.5% of the cases; moreover, it is known to be highly correlated with Epstein–Barr virus-related gastric cancer [14]. These findings suggest that minor carcinogenic drivers, such as *BRAF* mutations, might correlate with uncommon oncogenic pathways in gastric cancer.

*BRAF* mutations are more common in colorectal cancer cases with mismatch repair gene abnormalities, but not in gastric cancer [35]. In this study, the *BRAF* mutation-positive cases exhibited a gastric mucin phenotype. In serrated colorectal cancer, *BRAF* mutations are known to correlate with CDX2 inactivation, leading to the expression of the gastric phenotype [36]. It is possible that the *BRAF* mutation in HPAGC might counteract the expression of the intestinal phenotype.

The p53 mutation is common in HPAGC [37,38] and is thought to result in decreased expression of p21 (*WAF1/CIP1*; *CDKN1a*) and activation of cyclin D1 [39]. In the current study, abnormal accumulation of p53 protein was observed in all five cases of HPAGC. According to the subtype classification of gastric cancer due to molecular abnormalities as proposed by TCGA, many p53 mutations are found in the chromosomal instability subtype, which often exhibits an intestinal phenotype [14].

In this study, the HPAGC cases demonstrated a gastric phenotype and mutations of p53 and BRAF, as well as a possible association with chronic inflammation caused by mechanical stimuli, but no association with *H. pylori* infection. These characteristics suggest that HPAGC characterized by molecular abnormalities might comprise a new subtype with a distinct oncogenic background, albeit a minor population. All polyps analyzed in this study were very early lesions. Although it has been reported that BRAF mutations are more common in advanced stages [40], few studies have focused on early stage cancer. Genome instability (such as changes in gene copy numbers, rearrangements, and mutations) promotes clonal evolution of cancer cells through the accumulation of driver abnormalities [41]. It cannot be ruled out that *BRAF* mutations found in early stage cancer might be replaced by new, more powerful drivers as the cancer progresses.

In this study, we analyzed HPAGC cases with a focus on GHPs. Although the number of target cases was 102, which is not large compared to that of previous studies, five cases of HPAGC were found. The proportion of GHPs which were associated with cancer among our cases was similar to that reported in previous studies. It should be emphasized that *BRAF* mutations were found in two of five cases. This is a high frequency compared to previous studies on non-GHP-associated gastric cancer, but the small population of five cases requires further accumulation of cases. This finding suggests that a rare *BRAF* gene mutation might specifically accumulate in GHPs, even though they exhibit a low carcinogenic frequency. These findings provide new insight into HPAGC that is worth investigating in a larger cohort.

*BRAF* mutations are associated with poor prognosis in metastatic colorectal cancer [42], but treatment with drugs such as vemurafenib, which targets *BRAF* mutations, has demonstrated high efficacy in melanoma patients [43]. The results of this study reaffirm the significance of *BRAF* mutations as oncogenic drivers in gastric cancer and highlight the need to investigate HPAGC in a large number of GHP cases to determine the clinical significance of our findings.

## 4. Materials and Methods

### 4.1. Patients

We analyzed a total of 102 patients with GHPs who were subjected to endoscopic resection at the Miyoshi Central Hospital and histopathologically diagnosed by the Department of Molecular Pathology, Nara Medical University, Japan, between 2013 and 2019. As written informed consent was not obtained from the patients for their participation in the present study, all identifying information was removed from patient samples prior to their analysis to ensure strict privacy protection (unlinked anonymization). All procedures were performed in accordance with the Ethical Guidelines for Human Genome/Gene Research enacted by the Japanese Government and with the approval of the Ethics Committee of Nara Medical University, Japan (approval number, 937, 20 October 2014).

The endoscopic examination was performed by the two co-authors (TM and KN) using a GIF-H260 or GIF-H260Z endoscope (Olympus Optical Co., Ltd., Tokyo, Japan) under white light observation, contrasted chromoendoscopy, and narrow band imaging.

### 4.2. Histological Analyses

Histological evaluation was performed using hematoxylin and eosin staining. Histological atypia was evaluated as follows: low grade, mild to moderate nuclear swelling; high grade, moderate to marked nuclear swelling, nuclear pseudostratification, and weakened mucus production; and cancer, structural atypia, and/or invasion, in addition to alterations similar to high-grade lesions. *H. pylori* infection and intestinal metaplasia were evaluated according to the updated Sydney classification [15]. Each grade (none, mild, moderate, and severe) was quantified as 0, 1, 2, and 3, respectively, and statistically analyzed. PAS staining grades (0, 0.5, 1, 2, and 3) were classified by the ratio of the area of the PAS-positive region in the cells: 0, 0%; 0.5, <10%; 1, 10–25%; 2, 25–50%; and 3, >50%. Granulation tissue grade was evaluated by the area occupied by the polyp: 0, <10%; 1, 10–50%; 2, 50–75%; and 3, >75%.

### 4.3. Immunohistochemistry

Consecutive 4 μm thick sections were immunohistochemically stained using the immunoperoxidase technique described previously [44], with the listed primary antibodies (Table 4) and appropriate secondary antibodies (Medical and Biological Laboratories, Nagoya, Japan) (all 0.2 µg/mL). The tissue sections were then color-developed with diamine benzidine hydrochloride (Dako, Glostrup, Denmark) and counterstained with Meyer’s hematoxylin (Sigma-Aldrich Chemical Co., St. Louis, MO, USA). Staining indices were evaluated by examining 1000 epithelial cells, and the frequency of positive nuclear staining was determined. For p53 and CDX2, an index of more than 10% was considered to represent a “positive case”.

### 4.4. Enzyme-Linked Immunosorbent Assay (ELISA)

In samples of polyp specimens, granulation tissues were manually dissected under a microscope using sterile needles from four consecutive 8 μm thick sections of formalin-fixed and paraffin-embedded blocks. For deparaffinization, 1 mL of xylene was added to the cells in 1.5 mL microcentrifuge tubes and mixed well using a vortex mixer for at least 5 min, followed by two washes with 1 mL 100% ethanol. Proteins were then extracted by lysis with a RIPA buffer containing 0.1% SDS (Thermo Fisher, Tokyo, Japan). An ELISA kit (Abcam, Cambridge, MA, USA) was used to measure the concentration of 4-HNE. The assay was performed according to the manufacturer’s instructions, and whole-cell lysates were used for the measurements.

### 4.5. Mutation Analysis

Five consecutive 8 μm thick sections from formalin-fixed and paraffin-embedded blocks of polyp tumor specimens were analyzed for the presence of gene mutations in *BRAF* exon 15 and *KRAS* codons 12 and 13. The samples were sent to SRL (Tokyo, Japan) for mutation screening, which was performed using the Luminex (xMAP) assay [45].

### 4.6. Statistical Analyses

Statistical significance was calculated using a two-tailed Fisher’s exact test and ordinary ANOVA, using the InStat software (GraphPad, Los Angeles, CA, USA). Regression analysis was performed using the Pearson’s regression test. Statistical significance was set at a two-sided *p* value < 0.05.

## Figures and Tables

**Table 1:** Relationship between polyp atypia and *H. pylori* infection or proliferation.

Parameter	Polyp Atypia ^(1)^				*p*-Value
	None	Low Grade	High Grade	Cancer	
Number	72	20	7	5	
Age (yrs)	55 ± 13	60 ± 11	63 ± 10	70 ± 5	0.0191
Sex (male: female)	22:22	12:8	4:3	3:2	NS
Size (mm)	4.7 ± 1.8	6.9 ± 1.7	12.3 ± 5.9	14.6 ± 5.4	<0.0001
*H. pylori* infection					
Incidence (%)	9	10	0	20	NS
Grade ^(2)^	0.18 ± 0.65	0.10 ± 0.31	0	0.20 ± 0.45	NS
Intestinal metaplasia ^(2)^					
Incidence (%)	32	55	29	40	NS
Grade ^(2)^	0.5 ± 0.88	0.55 ± 0.51	0.29 ± 0.49	0.40 ± 0.55	NS
PAS staining ^(3)^	2.0 ± 0.1	1.4 ± 0.5	0.8 ± 0.3	0.6 ± 0.2	<0.0001
Ki-67 index (%) ^(4)^	26 ± 13	52 ± 23	74 ± 18	86 ± 10	<0.0001

^(1)^ Low grade, mild to moderate nuclear swelling; high grade, moderate to marked nuclear swelling, nuclear pseudostratification, and weakened mucus production; cancer, structural atypia, and/or invasion in addition to alterations similar to high-grade lesions. ^(2)^ According to the updated Sydney classification [15], each grade (none, mild, moderate, and severe) was quantified as 0, 1, 2, and 3, respectively, and the results were statistically analyzed. ^(3)^ PAS staining grades were classified according to the ratio of the area of the PAS-positive region in the cells: 0, 0%; 0.5, <10%; 1, 10–25%; 2, 25–50%; 3, >50%. ^(4)^ Ki-67 staining was examined in 1000 epithelial cells and the frequency of positive nuclear staining was determined.

**Table 2:** Relationship between atypia and granulation tissue, oxidative stress, or stemness.

Parameter	Polyp Atypia ^(1)^				*p*-Value
	None	Low Grade	High Grade	Cancer	
Number	44	20	7	5	
Granulation tissue (%) ^(2)^	15 ± 12	48 ± 23	79 ± 7	87 ± 7	<0.0001
Tumor 8-OHdG index ^(3)^	23 ± 12	52 ± 18	86 ± 8	94 ± 5	<0.0001
4-HNE (ng/g) ^(4)^	0.5 ± 0.1	0.8 ± 0.2	1.8 ± 0.3	2.4 ± 0.3	<0.0001
CDX2 incidence (%) ^(3)^	25	50	29	40	NS
p53 incidence (%) ^(3)^	0	0	43	100	0.0075
NS index (%) ^(3)^	18 ± 8	53 ± 17	80 ± 2	84 ± 6	<0.0001

^(1)^ Low grade, mild to moderate nuclear swelling; high grade, moderate to marked nuclear swelling, nuclear pseudostratification, and weakened mucus production; cancer, structural atypia, and/or invasion in addition to alterations similar to high-grade lesions. ^(2)^ Evaluated according to the area occupied by the polyp: 0, <10%; 1, 10–50%; 2, 50–75%; 3, >75%. ^(3)^ For assessment of these parameters, 1000 epithelial cells were examined, and the frequency of positive nuclear staining was determined. For p53 and CDX2, cases were judged as positive when the frequency of positive cells (index) was 10% or more. ^(4)^ 4-HNE levels in extracts from granulation tissues were measured using ELISA.

**Table 3:** Adenocarcinoma cases in hyperplastic polyps.

	Case				
	1	2	3	4	5
Polyp size (mm)	11	8	20	20	14
Cancer lesion (mm)	2	4	3	3	2
Histology ^(1)^	tub1	Pap + tub1	tub1	tub1	Pap + tub1
Invasion	In situ	In situ	Invasive	In situ	In situ
Mucin type ^(2)^	Mixed	Gastric	Gastric	Mixed	Mixed
Ki-67 index (%)	94	92	85	70	90
8-OHdG index (%) ^(3)^	100	100	92	88	90
CDX2 index (%) ^(3)^	24	0	0	5	80
p53 index (%) ^(3)^	78	72	19	17	89
NS index (%) ^(3)^	78	87	85	82	88
*KRAS* G12D/G13D	-/-	-/-	-/-	-/-	-/-
*BRAF* V600E	-	+	+	-	-

^(1)^ Histological classification was based on the Japanese Gastric Cancer Classification guidelines [16]. Pap, papillary adenocarcinoma; tub1, well-differentiated tubular adenocarcinoma. ^(2)^ Mixed, MUC5AC+/MUC2+; gastric, MUC5AC+/MUC2-. ^(3)^ These parameters were determined by examining 1000 epithelial cells and recording the frequency of positive nuclear staining.

**Table 4:** Antibodies used for immunohistochemistry.

Target ^(1)^	Manufacturer ^(2)^	Clone	Code	Working Concentration (μg/mL)
Ki-67	DAKO-Agilent	MIB-1	M7240	0.5
8-OHdG	JaIKA	N45.1	MOG-100P	5
CDX2	Abcam	CDX2-88	ab157524	0.2
p53	Abcam	PAb240	ab26	1
NS	Abcam	-	ab70346	0.5
MUC5AC	DAKO-Agilent	CLH2	M731601	0.5
MUC2	DAKO-Agilent	Ccp58	M7313	0.5

^(1)^ 8-OHdG, 8-hydroxy-2′-deoxyguanosine; CDX2, caudal type homeobox transcription factor 2; NS, nucleostemin. ^(2)^ DAKO-Agilent, Santa-Clara, CA, USA; JaIKA, Fukuroi, Japan; Abcam, Cambridge, UK.

## Data Availability

Not applicable.

## Abbreviations

GHP	gastric hyperplastic polyps
CDX	caudal type homeobox transcription factor
HPAGC	hyperplastic polyp-associated gastric cancer
BRAF	v-raf murine sarcoma viral oncogene homolog B1
AKT	v-akt murine thymoma viral oncogene homolog
TCGA	the cancer genome atlas
PAS	periodic acid–Schiff
HNE	hydroxy nonenal
8-OHdG	8-hydroxy-2′-deoxyguanosine
NS	nucleostemin
PIK3CA	phosphatidylinositol 3-kinase catalytic subunit alpha
